# Reducing malnutrition in Cambodia. A modeling exercise to prioritize multisectoral interventions

**DOI:** 10.1111/mcn.12770

**Published:** 2020-08-24

**Authors:** Arnaud Laillou, Ludovic Gauthier, Frank Wieringa, Jacques Berger, Samnang Chea, Etienne Poirot

**Affiliations:** ^1^ United Nations Children's Fund (UNICEF), Maternal, Newborn and Child Health and Nutrition section Phnom Penh Cambodia; ^2^ Independent consultant Phnom Penh Cambodia; ^3^ Institute of Research for Development (IRD), UMR Nutripass IRD‐UM2‐UM1 Montpellier France; ^4^ Council of Agriculture and Development (CARD), Office of the Council of Ministers Phnom Penh Cambodia

**Keywords:** Cambodia, children under 5 years of age, integrated approach, modeling, stunting, wasting

## Abstract

Undernutrition is a major contributor to child morbidity and mortality and poses a large burden to the economy, in Cambodia. This study explored factors contributing to child stunting and wasting and their regional inequalities among 1,938 Cambodian children aged 6–23.9 months. Data were drawn from a longitudinal study (year 2017) conducted in six districts of two north‐eastern provinces and the capital and used as cross‐sectional. Socio‐demographic and household characteristics, children's feeding practices during the previous 24 hr, and children's length and weight measurements were collected. Gradient boosting models were used to calculate the contribution of determinants to child undernutrition whereas concentration index was used to assess the impact of those determinants on stunting and wasting inequalities among socioeconomic groups. It was found that low‐household wealth could predict 21% to 45% of child stunting and 23% to 36% of wasting across regions. After wealth, source and treatment of drinking water were found the second major predictor for stunting (15%) and wasting (21%). Combining child nutrition and household water, sanitation and hygiene indicators predicted around 30% of child undernutrition, either in the form of stunting or wasting. Mothers' education predicted >30% of stunting in the north‐eastern region. Results highlight that a complex interplay of factors contributes to child stunting and wasting. An integrated, intersectoral, equity‐focused approach that addresses children's dietary quality, household's water, sanitation and hygiene conditions, mother's education, and poverty is likely to yield the highest impact in achieving further gains in nutritional status among Cambodian children.

**Key messages**

- Household wealth status was found to be the strongest independent predictor of child malnutrition (stunting and wasting). Interventions that target bottom wealth inequality may be most effective to reduce the 23% of stunting inequality found between socioeconomic groups.
- Poor water, sanitation, and hygiene (WASH) indicators and a low child feeding index score were found equally predictive for 30–40% of stunting and wasting prevalence. These findings suggest a need for strategic collaboration between the two sectors to further reduce child wasting and stunting, in Cambodia.
- Multisectoral engagement through integrated and complementary approaches is likely to yield greater efficiency to further improve nutritional status in Cambodian children.

## 1 INTRODUCTION

Approximately 40% of children under 5 years of age in low‐income and middle‐income countries are at risk of not reaching their full developmental potential; undernutrition is a major contributor to this loss of human capital (McCoy, Peet, & Ezzati, 2016).

In Cambodia, despite substantial progmaternal child nutrition resses made towards reducing maternal and child mortality (Greffeuille et al., 2016; National Institute of Statistics, 2015), undernutrition remains a persistent challenge with 32% of the children under 5 years of age being stunted and almost 10% wasted. The high prevalence of stunting and the large proportion of severely wasted children who are not receiving therapeutic treatment (approximately 94% of the children affected, in 2017) add to the burden of morbidity and mortality in the under‐five population (Khara & Dolan, 2014). The economic burden of undernutrition among women and children in the country is estimated at USD 145–266 million annually; 45% of these economic losses are linked to stunting (low length‐for‐age), and approximately 3% are associated with wasting (low weight‐for‐length; Moench‐Pfanner et al., 2016).

There is increasing recognition that the current nutrition‐focused approach will not be sufficient to achieve global targets for improvements in maternal and child nutrition (International Food Policy Research Institute, 2016). Multisectoral engagement around common goals of improved child survival, growth, and development through integrated and complementary approaches is widely considered to yield greater efficiency of investment (Irwin, Siddiqi, & Hertzman, 2007; Samson, Fajth, & Francois, 2016).

This paper analyses key drivers of child stunting and wasting and explores the contribution of several Integrated Early Child Development (IECD) factors on socioeconomic inequities of child's stunting and wasting, in Cambodia. Data were drawn from a longitudinal study (2017 data) part of the “Myhealth” project under the UNICEF‐Cambodia IECD programme in collaboration with provincial departments of the Ministry of Health.

## 2 METHODS

### 2.1 Data sources

Interviews with mothers (caretakers) of children under the age of three were conducted in the capital, Phnom Penh (Russei Kaev district) and two north‐eastern provinces, Kratie (Chitr Borie and Krong Kratie districts) and Ratanakiri (Ou Chum, Krong Ban Lung and Bar Kaev districts) as part of a longitudinal study under the project called MyHealth. The main objective of the project was to collect in‐depth data over a 3‐year period on the health and nutritional status of children under the age of 5 years and their mothers in selected districts in three provinces to better inform the government on progress that can be made with enhanced health monitoring. Study sites included urban, semiurban, rural, and remote areas. Russei Kaev district in Phnom Penh is situated on the boarder of the Mekong river and houses a large migrant and poor population from mixed ethnic origins. Access to services is limited, yet income generation opportunities are diverse, and coverage of educational facilities is good. In Kratie, most of the population is ethnic Khmer (Cambodian, 80%). The Mekong River provides the opportunity to farm and produce crops. Four out of five residents are employed in agriculture. Beside the urbanised district of Banlung, Ratanakiri is formed by rural settlements where most inhabitants are smallholder farmers who practice a subsistence agriculture supplemented by some food collection from surrounding forests and rivers. Ratanakiri has a large proportion of indigenous people with a high number of ethnic groups that have their own language and culture and live in remote areas.

A sample size of 1,200 children under 3 years of age per site was calculated required to observe a reduction in child stunting from 32% to 26% over a 3–5‐year period (with a precision of 3% and a dropout of 20%). A list of all children under 3 years of age living in the 169 villages included in the study was obtained from midwives and village health volunteers. This paper uses data of the households with children between 6 and 23.9 months (*n* = 1,938).

An electronic tablet‐based questionnaire was used to collect information on (a) socioeconomic status, (b) health knowledge, (c) diet, including dietary diversity of children and women and breastfeeding practices, (d) WASH practices, and (e) mother and child anthropometry. Baseline data were collected in April–May 2016, followed by six consequent follow‐ups that will run until March 2019. Data for children collected at follow‐up visit 2 (October–November 2017) were used for the present study.

### 2.2 Ethical approval

Ethical approval for the study was obtained from the Cambodia National Ethical Committee for Health Research. Informed consent was obtained from all participants, with consent obtained from parents or guardians for participating children.

### 2.3 Anthropometric measurements

Gender, weight, length, and middle upper arm circumference (MUAC) were recorded for all children. Field workers measured length using UNICEF length boards with standing plates and moveable head boards accurate to 1 mm. We calculated length‐for‐age *z* scores and weight‐for‐length *z* scores using World Health Organization (WHO) 2006 standards for children 0–59 months (de Onis, 2006) using WHO Anthro software (version 3.2.2, January 2011). Field workers measured weight using calibrated digital balances (SECA) with 100‐g precision. The device used to measure the MUAC of children was a plastic, coloured, insertion tape (UNICEF supply division in Copenhagen) marked in millimetres, with cut‐off points from red to yellow at 115 mm and from yellow to green at 125 mm, incapable of stretching and unresponsive to temperatures. Length‐for‐age *z* scores < −2 standard deviation (SD) was defined as stunting whereas weight‐for‐length *z* scores < −2 *SD* and/or MUAC below 125 mm or presence of oedema was defined as wasting.

### 2.4 Feeding practices

Infant and young child feeding practices were assessed per UNICEF and WHO indicators for appropriate breastfeeding and complementary feeding practices (WHO, 2008), including continued breastfeeding, bottle feeding, meal frequency, and dietary diversity in the previous 24 hr. Continued breastfeeding, as binary variable, denoted if the child was being breastfed until the age of 23.9 months. Bottle feeding was recorded as a binary variable, denoting if the child received bottle feeding. Dietary diversity was based on consumption of foods from the following groups: (a) grains, roots, tubers, (b) legumes and nuts, (c) vitamin A fruits and vegetables, (d) other fruits and vegetables, (e) meats, (f) eggs, and (g) dairy products (WHO, 2008). Minimum meal frequency is defined as 2 for 6–8 months child breastfed, 3 for 9–23 months child breastfed, and 4 for nonbreastfed child 6–23 months (WHO, 2008). This indicator is intended as a proxy for energy intake from foods other than breast milk. An adapted child feeding index (Ruel & Menon, 2002) combining the four behaviours above (continued breastfeeding, use of bottle, dietary diversity, and meal frequency) was created for 6–8 months, 9–11 months, and 12–23 months by giving scores from 0 to 7 according to the practices claimed (Table S1). The child feeding index was then divided into three groups according to the score obtained: (1–3) low child feeding index score, (4–5) medium child feeding index score, and (6–7) high child feeding index score.

### 2.5 WASH indicators

WASH variables included type of water used, type of sanitation, and treatment of the water. Although the treatment was a binary variable (adequate/not adequate), the other two were classified into improved or not according to the classification used during national surveys, such as the Cambodian Demographic Health Survey (National Institute of Statistics, 2015).

### 2.6 Variables included in the analyses

The child's age in months was calculated by subtracting the date of the visit from the date of birth of the child; results were classified in three groups: (6–12 months), (12–18 months), and (18–23.9 months). Gender was considered as a binary variable. Household wealth index, a composite measure of a household's living standard, was constructed using principal component analysis, as described by Filmer and Pritchett (Filmer & Prichett, 2001). The index includes materials used for housing construction, type of cooking fuel, and ownership of selected assets, such as radio, television, and refrigerator. WASH indicators (source of drinking water and type of sanitation facility) were not included (O'Donnell, Van Doorslaer, Wagstaff, & Lindelow, 2008). Once the index was created, household was divided in quintiles (poorest, poor, middle, richer, and richest). Mother's education was divided in three groups: no education, if the mother had attended no schooling; informal or primary school only (primary); and higher education if otherwise (secondary and above). Finally, access to national health campaign and birth certification were considered as binary variables using yes if the child had respectively received vitamin A supplementation 1Vitamin A is integrated with other health activities such as vaccination over the last year during the campaign and/or had a birth certificate.

### 2.7 Statistical analysis

To rank the predictive power of different variables 2Low wealth quintile, low mother education, no access to national health campaign, access to nonimproved sanitation facility, no adequate treatment of water, access to nonimproved source of drinking water, low child feeding index score, and no birth registration on stunting and wasting, we used Boosted Regression Trees (BRTs) methods. BRT is a tree‐based method and differs fundamentally from traditional regression techniques. In broad strokes, boosted regression trees combine the strength of two algorithms methods: regression trees (models that relate a response to their predictors by recursive binary splits) and boosting (a method that combines simple models to give improved predictive performance; leathwick, Elith, Chadderton, Rowe, & Hastie, 2006). This method has important advantages, as it can handle different types of predictor's variables. In addition, it can model complex nonlinear relationships, and its predictive performance is superior to most traditional modeling methods (Elith, Leathwick, & Hastie, 2008). Although BRTs are complex, the results can be summarized through relative influences of the predictor variables on the outcome that allows to rank the determinant included in the model per their predictive power. Using the formulae developed by Friedman (Friedman, 2001), the influence of each determinant is scaled so that the sum adds to 100, with higher numbers indicating stronger influence on the response.

Then, the current paper explored the contribution of 10 variables to socioeconomic inequities on stunting and wasting in six districts of Cambodia. The inequality in stunting and wasting was calculated using the concentration index (referred below as CI; Wagstaff, van Doorslaer, & Watanabe, 2003). This approach of measuring inequality has been widely used and recognized as a standard tool (Emamian, Fateh, Gorgani, & Fotouhi, 2013; Kien et al., 2016; Rabbani, Khan, Yusuf, & Adams, 2016). The concentration index can be computed as twice the covariance of the health variable under study and a person's relative rank in terms of economic status, divided by the health variable mean. The person's relative rank *ri* = *i*/*N* is a fractional rank of individual *i* in the living standards distribution, with *i* = 1 for the poorest and *i* = *N* for the richest. The value of this index ranges from −1 to +1: Its negative values imply that the variable is concentrated among disadvantaged people whereas its positive value implies the opposite. In the case of no inequality, the concentration index is equal zero.

All analyses were performed using STATA software version 13.1 (College Station, TX: StataCorp) and R software version 3.4.0.

## 3 RESULTS

Among the 1,938 children aged 6–23 months, 51.4% were female. General characteristics of the sample are represented in Table 1. The prevalences of stunting and wasting were significantly different between the selected provinces (*p* < 0.001): respectively 14.6% and 9.2% in Phnom Penh and 26.5% and 15.2% in the north‐eastern districts. The type of sanitation was significantly different between Phnom Penh and the north‐eastern region. Almost two third of the children in Phnom Penh were living in household with access to improved sanitation compared with less than one third in the north‐eastern study locations.

**Table 1:** Characteristics of the sample

	North‐eastern (*N* = 1,364)				Phnom Penh (*N* = 574)			
Characteristic	*N*	%	95% confidence of interval		*N*	%	95% confidence of interval	
Child age (months)								
(6–12)	400	29.3	27.0	31.8	145	25.3	21.9	29.0
(12–18)	519	38.1	35.5	40.7	231	40.2	36.3	44.3
(18–23.9)	445	32.6	30.2	35.2	198	34.5	30.7	38.5
Gender								
Female	703	51.3	48.6	53.9	294	49.6	44.7	52.9
Male	668	48.7	46.1	51.4	280	50.4	47.1	55.3
Mother's education								
No education	421	30.9	28.5	33.4	85	14.8	12.1%	18.0
Primary	546	40.0	37.5	42.7	204	35.5	31.7%	39.6
Secondary and above	397	29.1	26.8	31.6	285	49.7	45.6%	53.7
Household wealth quintile								
Poorest	372	27.3	25.0	29.7	23	4.0	2.7	6.0
Poor	355	26.0	23.8	28.4	49	8.5	6.5	11.1
Middle	332	24.3	22.1	26.7	134	23.3	20.1	27.0
Rich	163	12.0	10.3	13.8	133	23.2	19.9	26.8
Richest	142	10.4	8.9	12.1	235	40.9	37.0	45.0
Child feeding index (CFI)								
Low CFI score	515	37.8	35.2	40.4	128	22.3	19.1	25.9
Medium CFI score	683	50.1	47.4	52.7	358	62.4	58.3	66.3
High CFI score	166	12.2	10.5	14.0	88	15.3	12.6	18.5
Have child's birth certificate								
No	477	35.0	32.5	37.5	104	18.1	15.2	21.5
Yes	887	65.0	62.5	67.5	470	81.9	78.5	84.8
Access to national health campaign								
No	369	27.1	24.8	29.5	110	19.2	16.1	22.6
Yes	995	73.0	70.5	75.2	464	80.8	77.4	83.9
Sanitation facility								
Nonimproved	921	67.5	65.0	70.0	187	32.6	28.9	36.5
Improved	443	32.5	30.0	35.0	387	67.4	63.5	71.1
Treatment of water								
Not adequate	440	32.3	29.8	34.8	53	9.2	7.1	11.9
Adequate	924	67.7	65.2	70.2	521	90.8	88.1	92.9
Source of drinking water								
Nonimproved	566	41.5	38.9	44.1	4	0.7	0.3	1.8
Improved	798	58.5	55.9	61.1	570	99.3	98.2	99.7
Stunting								
No	1,002	73.5	71.0	75.7	490	85.4	83.7	88.0
Yes	362	26.5	24.3	29.0	84	14.6	12.0	16.3
Wasting								
No	1,157	84.8	82.8	86.6	521	90.8	88.1	92.9
Yes	207	15.2	13.4	17.2	53	9.2	7.1	11.9

The top contributing predictors of wasting and stunting among children 6–23 months are listed in Tables 2 and 3, respectively. Low‐wealth category was the main predictor of wasting in every district as it could predict three out of 10 wasted children. Combining WASH (sanitation, water access, and treatment of water) and child feeding indicators predicted about 32.9% of wasting in Phnom Penh and 41.4% in the north‐east. For stunting, similar ranking in the contributing predictors are shown in Table 3. Among all factors considered, the main predictor of stunting was lowest wealth quintile (21% to 45% depending on the province). WASH indicators and the child feeding index were equally important predictors in Phnom Penh (respectively 14.7% vs. 15.4%) and the north‐eastern districts (respectively 18.0% vs. 16.8%). In the north‐east, one out of three stunted children could be predicted by the low mother education factor.

**Table 2:** Relative importance of variables for the prediction of wasting among children 6–23.9 months (%)

Indicator	Phnom Penh (*n* = 574)	Kratie (*n* = 646)	Ratanakiri (*n* = 718)	North‐eastern^c^ (*n* = 1,364)
				
Low wealth quintile	29.8	35.7	22.8	30.1
No mother education	19.9	10.3	20.4	13.1
Low child feeding index score	21.4	15.1	14.3	16.2
No access to national health campaign^a^	8.6	13.6	11.5	11.5
No birth certificate	8.7	3.2	7.3	3.9
WASH indicators				
Access to nonimproved sanitation facility	5.9	5.9	14.5	14.3
No adequate treatment of water	5.6	4.2	3.7	3.3
Access to nonimproved drinking water^b^	NA	11.9	5.5	7.6

a The proxy used was access to vitamin A supplementation.

b No data available for nonimproved drinking water in Phnom Penh as >99% have access to an improved source.

c North‐eastern includes Kratie and Ratanakiri.

**Table 3:** Relative importance of variables for the prediction of stunting among children 6–23.9 months (%)

Indicator	Phnom Penh (*n* = 574)	Kratie (*n* = 646)	Ratanakiri (*n* = 718)	North Eastern^c^ (*n* = 1,364)
				
Low wealth quintile	32.3	45.2	21.4	26.5
No mother education	17.5	12.2	19.0	30.6
Low child feeding index score	15.4	22.3	18.8	16.8
No access to national health campaign^a^	5.8	8.5	15.8	5.4
No birth certificate	14.1	2.5	4.6	2.6
WASH indicators				
Access to nonimproved sanitation facility	8.3	2.3	1.9	2.6
No adequate treatment of water	6.4	1.2	10.2	4.1
Access to nonimproved drinking water^b^	NA	5.3	8.2	11.3

a The proxy used was access to vitamin A supplementation.

b No data available for nonimproved drinking water in Phnom Penh as >99% have access to an improved source.

c North‐eastern includes Kratie and Ratanakiri.

Table 4 presents results from the decomposition analysis for stunting. It shows how the various characteristics of respondents contributed to the inequality in child stunting in both north‐eastern area and Phnom Penh. The different calculation steps can be illustrated through using “improved sanitation facility” for stunting in the north‐east. The results of steps 1–3, which are the risky birth interval coefficient, its mean, and its concentration index are shown in Columns 2–4, respectively. Step (4) involves the calculation of the elasticity (0.01729, not shown in the table) obtained by multiplying the improved sanitation facility mean (0.325) and its coefficient (−0.059) and then dividing the result by the mean of Ln odds _stunting_ (−1.109). The number in the “Contribution to CI” column (0.005), is obtained by multiplying the elasticity (+0.01729) and the concentration index (CI; 0.307). The above‐mentioned column shows the contribution to CI of each determinant like improved sanitation facility to the socioeconomic inequality in Ln odds _stunting_. To quantify the corresponding percentage contribution to CI of improved sanitation facility (2.71%), that is, Step (5), its contribution to CI is divided by the concentration index (CI) of the Ln odds _stunting_ (0.196). This process was repeated for each of the other determinants.

**Table 4:** Decomposition of concentration index to estimate stunting of children 6–23.9 months' inequality between socioeconomic groups

	North‐eastern districts^a^					Phnom Penh district				
	Coefficient	Mean	Concentration index (CI)	Contribution to CI	Contribution to CI in %	Coefficient	Mean	Concentration index (CI)	Contribution to CI	Contribution to CI in %
Child age (months)					−2.03					−3.47
(6–12)	−0.789	0.293	−0.045	−0.009	−4.80	−0.927	0.253	−0.018	−0.002	−0.11
(12–18)	−0.318	0.381	0.050	0.005	2.77	−0.554	0.402	−0.001	−0.001	−3.36
(18–23.9)	Ref	‐	‐	‐		Ref	‐	‐	‐	
Gender					−0.82					4.31
Female	−0.197	0.513	−0.018	−0.002		−0.485	0.488	0.008	0.003	
Male	Ref	‐	‐	‐		Ref	‐	‐	‐	
Mother's education					18.56					19.47
No education	Ref	‐	‐	‐		Ref	‐	‐	‐	
Primary	−0.540	0.400	0.019	0.004	1.91	0.246	0.355	−0.152	0.007	10.42
Secondary and above	−0.520	0.291	0.240	0.033	16.65	−0.13	0.497	0.1682	0.006	9.05
Wealth quintile					61.45					67.54
Poorest	Ref	‐	‐	‐		Ref	‐	‐	‐	
Poor	−0.326	0.260	−0.194	−0.015	−7.57	−0.052	0.085	−0.836	−0.002	−2.91
Middle	−0.409	0.243	0.310	0.028	14.16	0.060	0.233	−0.517	0.004	5.64
Rich	−0.255	0.120	0.673	0.019	9.43	−0.477	0.232	−0.051	−0.003	−4.38
Richest	−1.060	0.104	0.897	0.089	45.43	−0.365	0.409	0.592	0.047	69.19
Child feeding index (CFI)^b^					−0.15					−2.00
Low CFI score	Ref	‐	‐	‐		Ref	‐	‐	‐	
Medium CFI score	−0.0370	0.501	−0.009	−0.0001	−0.07	0.166	0.624	0.003	−0.001	−0.22
High CFI score	0.0120	0.122	0.119	−0.0002	−0.08	0.658	0.153	0.023	−0.001	−1.78
Have child's birth certificate					−0.31					9.37
No	Ref	‐	‐	‐		Ref	‐	‐	‐	
Yes	0.012	0.650	0.082	−0.001		−0.563	0.819	0.026	0.006	
Access to national health campaign					6.86					−0.76
No	Ref	‐	‐	‐		Ref	‐	‐	‐	
Yes	−0.295	0.730	0.069	0.013		−0.095	0.808	−0.013	−0.001	
Sanitation facility					2.71					1.44
Nonimproved	Ref	‐	‐	‐		Ref	‐	‐	‐	
Improved	−0.059	0.325	0.307	0.005		−0.061	0.674	0.045	0.001	
Treatment of water					−0.95					4.10
Not adequate	Ref	‐	‐	‐		Ref	‐	‐	‐	
Adequate	0.031	0.677	0.098	−0.002		−0.383	0.908	0.015	0.003	
Source of drinking water^c^					14.67					
Nonimproved	Ref	‐	‐	‐		‐	‐	‐	‐	‐
Improved	−0.394	0.590	0.139	0.029		‐	‐	‐	‐	‐
Ln odds of stunting		−1.109	0.196	0.196 (total sum)			−1.874	0.068	0.068 (total sum)	

a North‐eastern includes Kratie and Ratanakiri.

b A child feeding index (Ruel & Menon, 2002) combining these four behaviours was created for 6–8‐month, 9–11‐month, and 12–23‐month age groups and then divided in three groups (see methodology section).

c No data available for nonimproved drinking water in Phnom Penh as >99% have access to an improved source.

This concentration index quantifies how the variables are unequally distributed by economic status. For example, in both areas (Phnom Penh and the north‐east), mother with higher education, access to improved sanitation, and/or appropriate treatment of water are all positively associated (CI > 0) with economic status rank, that is, concentrated among people of higher economic status. The positive concentration index in the north‐eastern region (0.1963) and in Phnom Penh (0.0681) and the negative log odd of stunting (−1.1092 in the North East and −1.8742 in Phnom Penh) indicate that children from poorer households had a higher probability of being stunted. The observed inequality in stunting is mainly explained by household economic status in the north‐east selected districts and Phnom Penh (61.45% and 67.54%, respectively), after controlling for potential confounding. Being from the highest quintiles influences most of the stunting inequity (45% in the north‐eastern districts and 69% in Phnom Penh). Mother's education was the second most important factor influencing the stunting inequality among socioeconomic groups in both regions (18.6% in the north‐east and 19.5% in Phnom Penh).

Due to a limited number of wasted children (11.9% of 574 children) in Phnom Penh, the decomposition of concentration was only performed for the north‐eastern districts (Table 5). According to the contribution to CI, in the North East, mother with higher education, access to improved sanitation, improved source of drinking water, and/or appropriate treatment of water and high child feeding index score are all concentrated among people of higher economic status. As it was observed for stunting, children from poorer households had a higher probability of being wasted. The largest contributions to inequality in wasting in the north‐east were attributable to household economic status (51.2%), access to improved sanitation (26%), and improved source of drinking water (20.5%). Furthermore, mother's education (14.2%) and access to national health campaign (7.2%) showed a considerable contribution to the measured inequality.

**Table 5:** Decomposition of concentration index of children 6–23.9 months to estimate wasting inequality between socioeconomic group^a^

	Wasting (North‐eastern)				
	Coefficient	Mean	Concentration index (CI)	Contribution to CI	Contribution to CI in %
Child age (months)					−0.25
(6–12)	0.089	0.2933	−0.0452	−0.0009	0.84
(12–18)	0.0805	0.3805	0.0498	0.0007	−1.09
(18–23.9)	Ref	‐	‐	‐	
Gender					1.33
Female	0.2044	0.5125	−0.0177	0.001	
Male	Ref	‐	‐	‐	
Mother's education					14.24
No education	Ref	‐	‐	‐	
Primary	−0.1442	0.4003	0.0193	0.0006	0.8
Secondary and above	−0.2691	0.2911	0.2395	0.0105	13.44
Wealth quintile					51.16
Poorest	Ref	‐	‐	‐	
Poor	−0.2222	0.2603	−0.1944	−0.0063	−8.06
Middle	−0.3586	0.2434	0.3096	0.0152	19.37
Rich	−0.15	0.1195	0.6728	0.0068	8.64
Richest	−0.4665	0.1041	0.8966	0.0244	31.21
Child feeding index (CFI)^b^					−3.32
Low CFI score	Ref	‐	‐	‐	
Medium CFI score	−0.1935	0.5007	−0.0086	−0.0005	−0.6
High CFI score	0.2613	0.1217	0.1192	−0.0021	−2.72
Have child's birth certificate					1.17
No	Ref	‐	‐	‐	
Yes	−0.0305	0.6503	0.0823	0.0009	
Access to national health campaign					7.2
No	Ref	‐	‐	‐	
Yes	−0.1986	0.7295	0.0694	0.0056	
Sanitation facility					25.96
Nonimproved	Ref	‐	‐	‐	
Improved	−0.3632	0.3248	0.307	0.0203	
Treatment of water					−18.06
Not adequate	Ref	‐	‐	‐	
Adequate	0.3806	0.6774	0.0977	−0.0141	
Source of drinking water					20.56
Nonimproved	Ref	‐	‐	‐	
Improved	−0.3535	0.585	0.1387	0.0161	
Ln odd of wasting		−1.7832	0.0782	0.0782	

a Due to limited number of wasted children in the district of Russey Keo in Phnom Penh, the decomposition of concentration analysis was only performed for the North‐eastern region (Kratie and Ratanakiri).

b A child feeding index (Ruel & Menon, 2002) combining these four behaviours was created for 6–8‐month, 9–11‐month, and 12–23‐month age groups and then divided in three groups (see methodology section).

Child feeding index was neither an influencer of the inequality for stunting nor for wasting between socioeconomic groups. Access to national health campaign influenced to approximately 7% of the wasting inequalities between socioeconomic groups in the north‐eastern districts.

## 4 DISCUSSION

This study presents key factors associated with wasting and stunting among Cambodian children aged 6–23 months. Also, it highlights the main contributors to inequalities of these two forms of child undernutrition between socioeconomic groups.

In our study, household wealth status was the strongest predictor of child stunting and wasting. The greater nutrition vulnerability of impoverished populations and the positive intergenerational impact of poverty reduction on nutrition are widely recognized (Aguayo, Badgaiyan, & Paintal, 2015; Kavosi et al., 2014; Pravana et al., 2017; Saxton et al., 2016; Tiwari, Ausman, & Agho, 2014; Torlesse, Cronin, Sebayang, & Nandy, 2016). Several studies suggest that socioeconomic development, indicated by the increase of household wealth index, contributed to the reduction of stunting over one to two decades in Brazil (Correia et al., 2014), Cambodia (Ikeda, Irie, & Shibuya, 2013), and Bangladesh (Rabbani et al., 2016). Our results indicate that socioeconomic inequalities are the most important factor‐driving inequities in stunting and wasting among infants and young children aged 6–23 months, in Cambodia. According to our findings, inequalities in stunting between socioeconomic groups could be partially reduced (23% in the north‐eastern region and 8.4% in Phnom Penh) if the poorest quintile would reach the middle quintile level. Social safety net programmes are considered effective for addressing economic inequities, alongside longer term interventions such as fair taxation and employment creation (Rabbani et al., 2016). Financial incentives such as cash transfers are increasingly being used as poverty reduction efforts to assist vulnerable populations in accessing better diets, health services, and improved living environments. Yet, in the Philippines, conditional cash transfers had a positive impact on severe stunting only (Kandpal et al., 2016). Given the low prevalence of severe stunting among the lowest quintile (14%; National Institute of Statistics, 2015), the impact of similar cash transfer interventions in Cambodia may be limited due to the low number of children potentially benefiting from it. Therefore, in the context of Cambodia, interventions may need to focus on the design of social safety nets that reduce inequities between wealth quintiles and ensure universal access to a minimum package of interventions.

Poor WASH indicators and a low child feeding index score were predictors of 30–40% for stunting and wasting prevalence; these findings suggest a need for more integration between nutrition and WASH for reducing child undernutrition, in Cambodia. In 2013, using data from the Demographic Health Surveys from 2000 to 2010, household wealth and sanitation were found the main predictors of stunting (Ikeda et al., 2013). The importance given to nutritional indicators in this latter paper could be discussed as selection was restricted to early initiation of breastfeeding, without considering dietary quality indicators of complementary feeding for children aged 6–23 months. In a multidimensional poverty analysis that used data from Cambodia's 2014 Demographic Health Survey, the authors showed that the poorest children had the highest rates of nutrition deprivation and stunting nationally; however, a significant proportion of children in the highest wealth quintiles were also found to be nutritionally deprived as indicated by a significant prevalence of stunting (Karpati, de Neubourg, Laillou, & Poirot, 2020). This finding could explain why the child feeding index, the composite measure of the appropriateness of IYCF practices, was not significantly associated with stunting and wasting inequality between socioeconomic groups in our study. Formative research in Cambodia indicated that most Cambodians, in any setting, consider rice nutritious enough to support children's weight gain (UNICEF, Helen Keller International, & National Nutrition Program, 2016). This belief continues to influence widespread inadequacy of feeding practices. WASH interventions have focused on improved sanitation, point‐of‐use water treatment, and maternal hand washing over the last decades. In 2014, Ngure et al. highlighted the importance to address the vectors of soil, poultry faeces, and infant foods (Ngure et al., 2014) and create a baby friendly environment. This may be a relevant approach for Cambodia where the presence of pigs near the main household dwelling was found to be a risk factor associated with Giardia duodenalis infection in young children and potentially stunting (Caron et al., 2018).

In our study, households in the north‐eastern region had much lower access to safe sources of drinking water and improved sanitation facilities, compared with households in Phnom Penh. Increased latrine coverage is generally believed to be effective for reducing exposure to faecal pathogens and preventing disease, but some studies believe that this outcome cannot be assumed (Clasen et al., 2014), and other factors may interfere. For example, in Chad, inappropriate cleaning processes of containers used for water transport in the household increased by two, the risk of being wasted (Marshak, Young, Bontrager, & Boyd, 2017). Ingestion of parasites through faecal‐contaminated food or water is often associated with increased incidence of diarrhoea (WHO, 2001) and increased incidence of wasting. In our study, the use of a nonimproved water source was a strong contributor to child stunting (14.7%) and wasting (20.6%) inequality between socioeconomic groups in the north‐eastern study locations. Addressing microbiology contamination of water at the point of use may be an important interim measure to improve safe drinking water. Recent analysis of water quality in the MyHealth project at the collection and consumption levels showed considerable levels of water quality deterioration at the household level with concerning high levels of Coliform and *Escherichia coli* at point of use. More than 80% of the water samples for use in children under 5 years of age had more than 10 colony forming unit (CFU) of coliform per 100 ml of water, and more than 30% had more than 10 CFU of *E*. *coli* per 100 ml of water at the point of use (Poirot et al., 2020). Without ensuring water safe quality at collection and consumption, it will be challenging to ensure an impact on the reducing‐related morbidity and linear growth. Protecting children from contaminated water, inappropriate hygiene practices, and impropriate sanitation through a specific “under 5” WASH package may reduce faecal exposure, morbidity episodes and improve the nutritional status of Cambodian children. Although our study suggests positive outcomes of an integrated approach to WASH and nutrition, two recently conducted randomized controlled trials in Bangladesh and Kenya demonstrated limited or no benefit to the integration of WASH interventions with nutrition (Luby et al., 2018; Null et al., 2018).

In our study, 31% of the mothers in the north‐eastern region and 15% in Phnom Penh had no formal education. Our finding that maternal education was an important contributor to child stunting (~19% in both regions) and wasting (14% in North Eastern region) is consistent with evidence showing that children of educated mothers are less likely to be undernourished (Delpeuch, Traissac, Martin‐Prevel, Massamba, & Maire, 2000). In Cambodia, maternal education is linked to increased wealth (Ministry of Health, PMNCH, WHO, World Bank, & AHPSR, 2015) and access to health services (Yanagisawa, Mey, & Wakai, 2004). Long‐term interventions to improve mother's education by better access to high school degrees through scholarship programmes may support the reduction of child malnutrition, in Cambodia, in a long term. The fact that Cambodian's 2014 Demographic Health Survey reports that the proportion of girls who completed secondary school in Phnom Penh and the north‐eastern region is below 6% and 3%, respectively (National Institute of Statistics, Directorate General for Health, & ICF International, 2015), indicates a major challenge for the country and a need for inclusion as a priority issue in Cambodia's development agenda. As shown in Table 4 and 5, 94% of the 14.2% of mother's education contribution to wasting inequality between socioeconomic groups is concentrated in mothers who reach secondary education and above. Similar findings are observed for stunting with most of the inequality between socioeconomic groups observed in Phnom Penh and the north‐east. Those results highlight the potential positive impact on child wasting and stunting of allowing adolescent girls to reach and complete secondary school. In Indonesia and Bangladesh, a high level of maternal education was associated with protective caregiving behaviours (Semba et al., 2008). Therefore, a higher level of education for adolescent should help promote gender equality and empower those girls, which will ultimately might contribute to the reduction of child malnutrition (Cunningham, Ruel, Ferguson, & Uauy, 2015).

Our findings are representative of the six selected districts form Kratie, Ratanakiri (the north‐east), and Phnom Penh only. To generalize these findings to Cambodia, a similar analysis could be done with data from the most recent national survey (2014 CDHS). Also, our model is considering a selection of factors (wealth quintile, mother education, access to national health campaign, sanitation facility and treatment of water, source of drinking water, child's age, gender, child feeding index, and birth registration) and the inclusion of additional indicators, such as maternal nutrition might further improve insights in the contribution of various factors on stunting and wasting.

## 5 CONCLUSION

Our study confirms the complex interplay of sectorial factors associated with undernutrition. Findings allow to formulate many recommendations for policy reinforcement and intervention prioritization, in Cambodia. The potential for intersectorial gains presents the need for strategic approaches and synergies to be built through a process of collaboration and coordination across sectors at the national and subnational levels. Nutrition‐specific and nutrition‐sensitive interventions are both and together the best leverage for promoting positive nutritional status. A whole‐child‐focused strategy in Cambodia is required that brings together food, health, water, and every other sector required for a sustainable food and nutrition forthcoming.

The determinants of malnutrition cover a broad variety of biological, social, cultural, economic, and morbidity factors. The influence of these factors can be used to guide the development of strategies of intervention for reducing child malnutrition. More research is required to support a prioritization of basic and underlying determinants to be addressed to tackle poor nutritional status in Cambodian children most efficiently. An integrated, intersectoral, equity‐focused approach that addresses children's dietary quality, household's water, sanitation and hygiene conditions, mother's education, and poverty is likely to yield the highest impact in achieving substantial nutrition gains among Cambodian children.

## CONFLICT OF INTEREST

The authors declare that they have no conflicts of interest. The opinions and statements in this article are those of the authors and may not reflect official policies or opinions of the organisations they belong to.

## CONTRIBUTIONS

AL, LG, and EP did the study design, data analysis, and interpretation, respectively. AL, LG, FW, JB, and EP draft the manuscript. All authors reviewed and approved the final manuscript.

## Supporting information

Table S1. Child feeding index

Figure S1. Decomposition of stunting and wasting inequality between socioeconomic groups in the North Eastern region and Phnom Penh
