# Supplementary material for: Reducing malnutrition in Cambodia. A modeling exercise to prioritize multisectoral interventions
Source: Matern Child Nutr. 2020 Aug 24;16(Suppl 2):e12770. doi: 10.1111/mcn.12770 (PMC7591311; doi:10.1111/mcn.12770)
Supplement: Supplementary file 1 — Table S1. Child feeding index [file MCN-16-e12770-s001.docx]

### Supplementary Table 1 – Child feeding index

|  |  | 6-8 months | 9-11 months | 12-23 months |
| --- | --- | --- | --- | --- |
| Continued breastfeeding | yes | 2 | 2 | 1 |
|  | no | 0 | 0 | 0 |
| Use of bottle | yes | 0 | 0 | 0 |
|  | no | 1 | 1 | 1 |
| Dietary Diversity (past 24 hours)^a^ | 0 | 0 | 0 | 0 |
|  | 1-3 | 1 | 1 | 1 |
|  | 4+ | 2 | 2 | 2 |
| Meal frequency (past 24 hours) | 0 meal/d | 0 | 0 | 0 |
|  | 1 meal/d | 1 | 1 | 0 |
|  | 2 meals/d | 2 | 1 | 1 |
|  | 3 meals/d | - | 2 | 1 |
|  | 4+ meals/d | - | 2 | 2 |
| TOTAL Score | | 7 | 7 | 6 |
| ^a^ Sum of (grains/tubers + meat/fish + eggs +legumes + beta-carotene rich foods+ other fruits/veg.) | | | | |
