# Supplementary material for: Reducing malnutrition in Cambodia. A modeling exercise to prioritize multisectoral interventions
Source: Matern Child Nutr. 2020 Aug 24;16(Suppl 2):e12770. doi: 10.1111/mcn.12770 (PMC7591311; doi:10.1111/mcn.12770)
Supplement: Supplementary file 2 — Figure S1. Decomposition of stunting and wasting inequality between socioeconomic groups in the North Eastern region and Phnom Penh [file MCN-16-e12770-s002.docx]

### Supplementary figure 1 – Decomposition of stunting and wasting inequality between socioeconomic groups in the Northeastern region and Phnom Penh^a^

^a^ Due to limited number of wasted children in the district of Russey Keo in Phnom Penh, the decomposition of concentration analysis was only performed for the North-eastern region (Kratie and Ratanakiri).
